# Genome-wide identification of quantitative trait loci and candidate genes for seven carcass traits in a four-way intercross porcine population

**DOI:** 10.1186/s12864-024-10484-y

**Published:** 2024-06-10

**Authors:** Huiyu Wang, Xiaoyi Wang, Yongli Yang, Yixuan Zhu, Shuyan Wang, Qiang Chen, Dawei Yan, Xinxing Dong, Mingli Li, Shaoxiong Lu

**Affiliations:** 1 Faculty of Animal Science and Technology, Yunnan Agricultural University, Kunming, Yunnan 650201 China; 2 Faculty of Animal Science, Xichang University, Xichang, Sichuan 615000 China

**Keywords:** GWAS, SLAF-seq, Pigs, Carcass traits, Candidate genes

## Abstract

**Background:**

Carcass traits are essential economic traits in the commercial pig industry. However, the genetic mechanism of carcass traits is still unclear. In this study, we performed a genome-wide association study (GWAS) based on the specific-locus amplified fragment sequencing (SLAF-seq) to study seven carcass traits on 223 four-way intercross pigs, including dressing percentage (DP), number of ribs (RIB), skin thinkness (ST), carcass straight length (CSL), carcass diagonal length (CDL), loin eye width (LEW), and loin eye thickness (LET).

**Results:**

A total of 227,921 high-quality single nucleotide polymorphisms (SNPs) were detected to perform GWAS. A total of 30 SNPs were identified for seven carcass traits using the mixed linear model (MLM) (*p* < 1.0 × 10^− 5^), of which 9 SNPs were located in previously reported quantitative trait loci (QTL) regions. The phenotypic variation explained (PVE) by the significant SNPs was from 2.43 to 16.32%. Furthermore, 11 candidate genes (*LYPLAL1*, *EPC1*, *MATN2*, *ZFAT*, *ZBTB10*, *ZNF704*, *INHBA*, *SMYD3*, *PAK1*, *SPTBN2*, and *ACTN3*) were found for carcass traits in pigs.

**Conclusions:**

The GWAS results will improve our understanding of the genetic basis of carcass traits. We hypothesized that the candidate genes associated with these discovered SNPs would offer a biological basis for enhancing the carcass quality of pigs in swine breeding.

**Supplementary Information:**

The online version contains supplementary material available at 10.1186/s12864-024-10484-y.

## Background

Carcass traits, such as dressing percentage (DP), number of ribs (RIB), skin thinkness (ST), carcass length (CL) and loin eye traits (LE), are important economic traits in pig production, and are also the main target traits for pig breeding and improvement. Recently, a study showed that the number of vertebrae was related to carcass length and meat production [1]. From the perspective of pork consumption and the economic value of vertebrae, sparerib is one of the most valuable parts of the pork carcass [2]. Because these carcass traits are controlled by multiple genes, their genetic basis is complex, and they are difficult to accurately measure for live pig, the improvement effect of conventional breeding methods is limited. With the rapid development of molecular markers and the completion of pig genome sequencing, molecular breeding has become the most promising way to improve carcass traits of pigs. In recent years, significant progress has been made in the quantitative trait loci (QTLs) and candidate genes for pig carcass traits. To date, a total of 30,480 QTLs associated with carcass and meat quality traits have been added to the pig QTL database (https://www.animalgenome.org/cgi-bin/QTLdb/index, accessed on 25 April 2023). These findings have considerably improved our knowledge of the genetic architecture of pig carcass traits. However, the fine mapping of QTLs and the discovery of new candidate genes still need to be strengthened, and the molecular genetic basis of some carcass traits of pigs is still unclear.

Genome-wide association study (GWAS) was used to identify SNPs associated with pig economic traits. In recent years, GWAS based on SNP array for carcass-related traits of pigs has identified many QTLs and candidate genes [3, 4]. Liu et al. [5] genotyped 576 Large White × Minzhu intercross pigs using the Illumina Porcine SNP60K Beadchip and performed GWAS for CL. GWAS results showed that a total of 31 genome-wide significant SNPs on SSC7 were detected to be associated with CL. Additionally, a total of 836 Duroc pigs were genotyped using the Illumina Porcine SNP60 K BeadChip. Furthermore, the study found that the vertnin gene (*VRTN*) was located on SSC7 at 103 Mb and was significantly associated with vertebrae number and carcass lengths [6]. Zhuang et al. [7] used Illumina Porcine SNP50K Beadchip to genotype 6,043 Duroc pigs and conducted a GWAS for loin muscle area (LMA) and loin muscle depth (LMD). Several QTLs and candidate genes related to LMA and LMD were identified. However, only a modest number of known SNPs could be found using GWAS based on the porcine SNP array. Furthermore, another genotyping technique employed recently was GWAS based on whole-genome sequencing (WGS). Nevertheless, GWAS for *Sus scrofa* (Sscrofa) based on WGS with large populations is still too expensive at the moment. Therefore, reduced representation sequencing technology known as specific-locus amplified fragment sequencing (SLAF-seq) was created to produce large-scale SNP data fast, reliably, efficiently, and inexpensively [8]. In contrast to SNP arrays, SLAF-seq technology, which is based on high-throughput sequencing, may produce millions of high-density SNP loci that cover the entire genome. Using SLAF-seq-based GWAS, several SNPs and potential genes have been found for a variety of economic traits in various animals [9–13]. SLAF-seq for pig genotyping effectively found numerous new mutation sites [14–16].

On 223 four-way intercross pigs raised in the same environmental conditions, seven carcass traits, DP, RIB, ST, carcass straight length (CSL), carcass diagonal length (CDL), loin eye width (LEW), and loin eye thickness (LET) were examined. Then, using SLAF-seq technology, GWAS was carried out to find significant SNPs linked to these traits. The findings provide a basis for pig breeding and the use of molecular markers to improve carcass quality.

## Results

### Phenotype description and correlation among growth traits

The statistical information on the seven carcass traits is shown in Table 1. The mean values for DP, RIB, ST, CSL, CDL, LEW and LET were 74.87%, 14.05, 3.47 mm, 96.86 cm, 83.44 cm, 8.21 cm and 6.17 cm, respectively. Coefficients of variation for the seven carcass traits were 3.87%, 4.12%, 32.45%, 5.97%, 7.52%, 11.28%, and 14.38%, respectively. The frequency distributions of these carcass traits are shown in Figure S1. These carcass traits appear to conform to the normal distribution.

The phenotypic correlation coefficients and genetic correlations for these carcass traits are shown in Table 2. The results revealed that CSL had the strongest positive correlation with CDL (*r* = 0.82, *p* < 0.001). LEW had the strongest negative correlation with LET (*r* = − 0.23, *p* < 0.001). The results of genetic correlations are similar to which of phenotypic correlations. There is a positive genetic correlation between CSL and CDL (*r* = 0.95 ± 0.02), and a negative genetic correlation between LEW and LET (*r* = − 0.59 ± 0.15).

**Table 1:** Descriptive statistics of seven carcass traits in the crossbred pigs

Trait	*N* ^a^	Min^b^	Max^c^	Mean	SD^d^	CV^e^
Dressing percentage, DP (%)	223	63.68	86.00	74.87	2.89	3.87
Number of ribs, RIB	211	12	15	14.05	0.58	4.12
Skin thickness, ST (mm)	223	1.30	8.90	3.47	0.11	32.45
Carcass straight length, CSL (cm)	223	79.00	117.00	96.86	5.78	5.97
Carcass diagonal length, CDL (cm)	223	63.00	101.00	83.44	6.27	7.52
Loin eye width, LEW (cm)	221	5.84	10.84	8.21	0.93	11.28
Loin eye thickness, LET (cm)	221	3.82	9.58	6.17	0.89	14.38

^a^ Number of samples

^b^ Minimum

^c^ Maximum

^d^ Standard deviation

^e^ Coefficient of variation

**Table 2:** Phenotypic correlation coefficients and genetic correlations among seven carcass traits in the crossbred pigs

Trait*	DP	RIB	CSL	CDL	ST	LEW
RIB	0.06 ^**a**^ 0.13 (0.19) ^**b**^					
CSL	–0.05 –0.11 (0.18)	–0.05 –0.08 (0.23)				
CDL	–0.17^*^ –0.29 (0.16)	–0.09 –0.14 (0.19)	**0.82** ^***^ **0.95 (0.02)**			
ST	–0.05 –0.06 (0.17)	0.04 0.15 (0.18)	0.03 0.02 (0.20)	0.15^*^ 0.27 (0.13)		
LEW	0.02 0.05 (0.16)	0.14 0.43 (0.22)	0.21^**^ 0.89 (0.26)	0.16^*^ 0.50 (0.21)	**–**0.05 –0.08 (0.19)	
LET	0.15^*^ 0.44 (0.27)	0.01 –0.04 (0.25)	0.05 0.20 (0.24)	–0.04 –0.03 (0.21)	**–**0.16^*^ –0.44 (0.25)	**–0.23** ^***^ **–0.59 (0.15)**

* DP, dressing percentage; RIB, number of ribs; ST, skin thickness; CSL, carcass straight length; CDL, carcass diagonal length; LEW, loin eye width; LET, loin eye thickness; ^*^ significant at *p* < 0.05, ^**^ significant at *p* < 0.01, ^***^ significant at *p* < 0.001. ^a^ Phenotypic correlation coefficient and ^b^ genetic correlation between traits. The numbers in brackets are standard errors. The maximum value and minimum value of the phenotypic correlation coefficient and genetic correlation between traits are in bold

### Identification of SLAFs and SNPs

In our previous study, 223 individuals were genotyped and descriptive statistics of the sequence data were presented [17–19]. In brief, the development of SLAF tags (314–344 bp sequence length) was accomplished using the restriction enzyme pair *Rsa*I and *Hae*III. The total number of paired-end reads obtained was 1109.92 Million. The average Q30 and GC content values were 90.74% and 44.83%, respectively (Table S1), showing the accuracy of our sequencing results for 223 accessions. With sequencing to an average depth of 11.94, a total of 1,552,377 SLAF tags (an average of 331,608 SLAFs for each individual) were identified from all individuals (Table S1). Additionally, Oryza sativa indica was employed as a control throughout the sequencing process. The results demonstrated that the normal construction of SLAF libraries was demonstrated by the enzyme digestion normally efficiency and paired-end comparison efficiency of control data, which were 90.77% and 95.4%, respectively.

After genomic mapping and SNP calling, a total of 16,997 polymorphic SLAF tags and 10,784,484 SNPs were obtained. After the genotyping results were filtered for a minimum MAF of 0.05 and locus integrity of 0.8, a total of 227,921 highly consistent SNPs were discovered. The density distributions of the SLAF, total SNPs and filtered SNPs across Sscrofa genome are shown in Figure S2. SNPs were found in almost all of the non-overlapping 1 Mb regions of the genome. The density distribution of total SNPs and filtered SNPs were calculated on each Sscrofa autosome and are shown in Table S2. The filtered SNP density across the 18 Sscrofa chromosomes was one SNP every 10.28 kb on average, which indicated that the data was reliable.

### Population structures, association analyses and identification of candidate genes

**Fig. 1:**
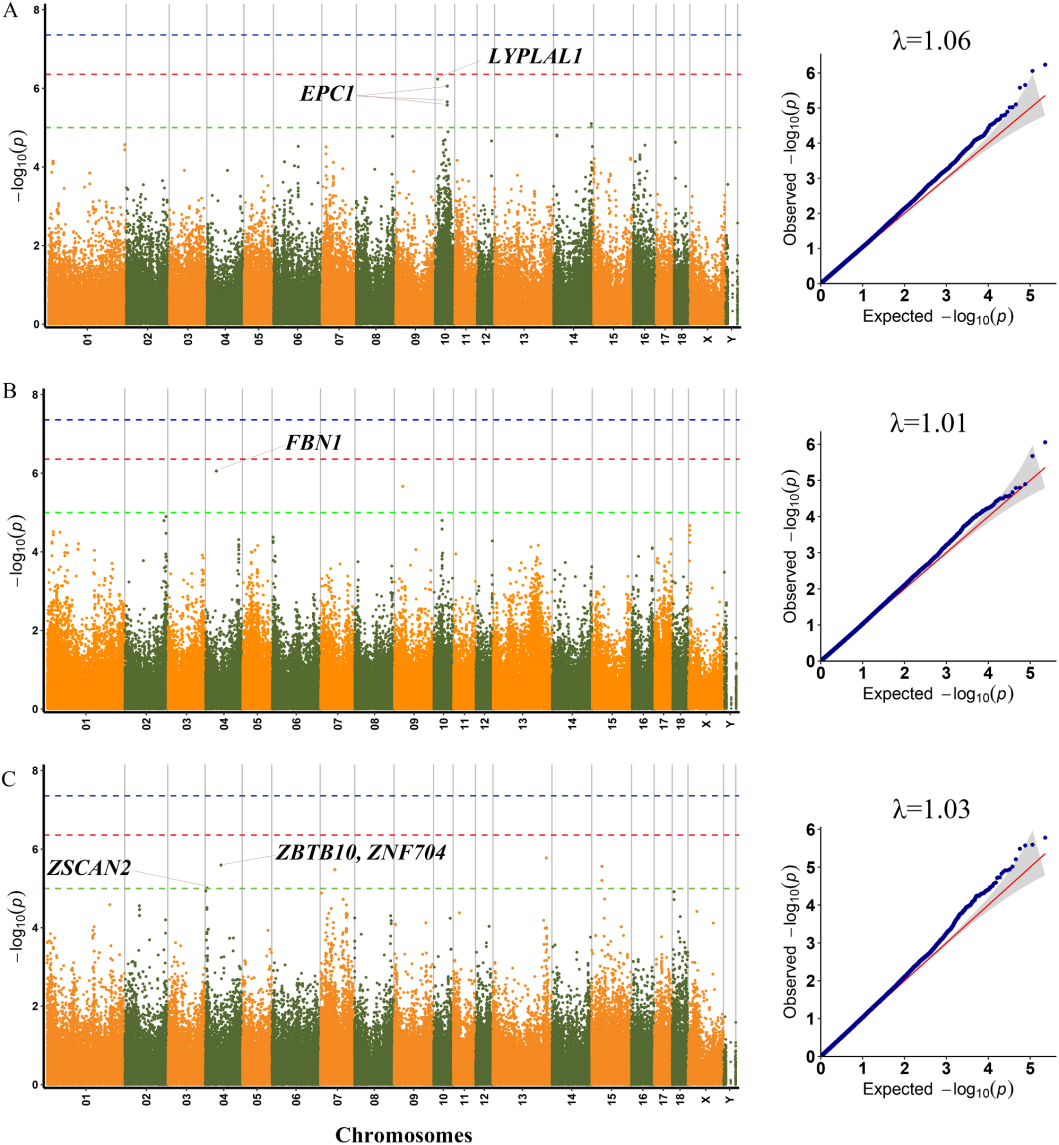
Manhattan plots and QQ plots for DP, RIB, and ST using MLM. (**A**) DP, (**B**) RIB, (**C**) ST. Negative log_10_*p*-values of the filtered high-quality SNPs were plotted against their genomic positions. The dashed lines of green, orange and blue correspond to the Bonferroni-corrected thresholds of *p* = 1.00 × 10^− 5^ (–log_10_*p* = 5), *p* = 4.39 × 10^− 7^ (–log_10_*p* = 6.36) and *p* = 4.39 × 10^− 8^ (–log_10_*p* = 7.36), respectively

**Fig. 2:**
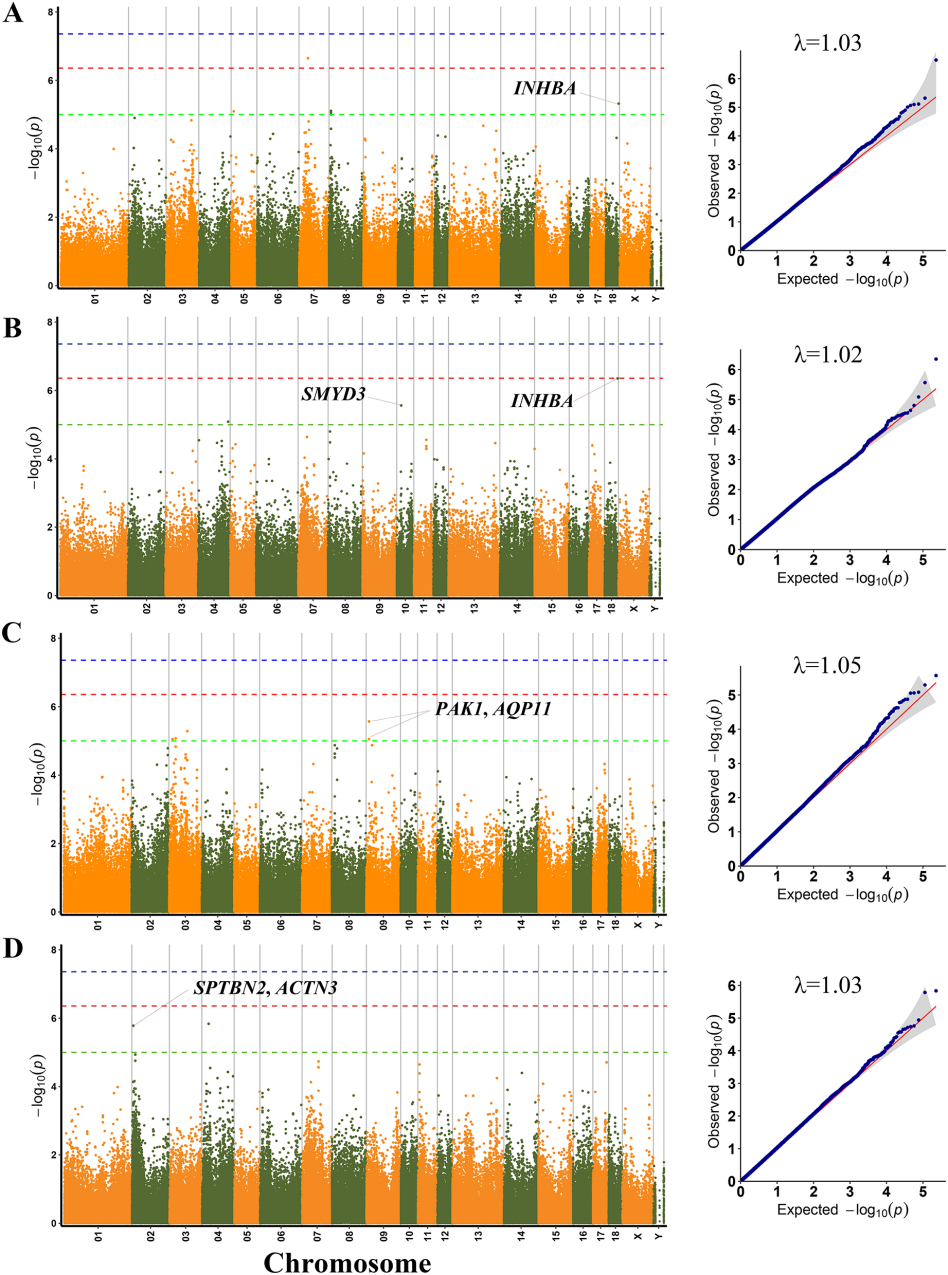
Manhattan plots and QQ plots for CSL, CDL, LEW and LET using MLM. (**A**) CSL, (**B**) CDL, (**C**) LEW, and (**D**) LET. Negative log_10_*p*-values of the filtered high-quality SNPs were plotted against their genomic positions. The dashed lines of green, orange and blue correspond to the Bonferroni-corrected thresholds of *p* = 1.00 × 10^− 5^ (–log_10_*p* = 5), *p* = 4.39 × 10^− 7^ (–log_10_*p* = 6.36) and *p* = 4.39 × 10^− 8^ (–log_10_*p* = 7.36), respectively

According to the population structure, the result was given by admixture with K from 2 to 10, where the optimal K was 9 (Figure S4). Since population stratification might affect GWAS, Q-Q plots of all traits were created. The observed –log10 p values calculated by the association study using MLM matched those that were anticipated, suggesting that the MLM did a good job of controlling for false positives. The Q-Q plot of each growth trait was displayed after the corresponding manhattan plot (Figs. 1 and 2). Using MLM, a total of 30 SNPs were found to be significant (*p* < 1.0 × 10^− 5^) for seven carcass traits (Table 3). The phenotypic variation explained (PVE) by the significant SNPs was from 3.37 to 16.17% (Table 3). Seven, two, six, six, three, five, and two SNPs were significantly associated with DP, RIB, ST, CSL, CDL, LEW and LET, respectively. These detected SNPs were distributed in twelve Sscrofa chromosomes (SSC), except for SSC1, SSC6, SSC11, SSC12, SSC16 and SSC17. Additionally, 38 genes total were thought to be prospective candidate genes and were situated 100 kb upstream and downstream of these significant SNPs (Table 3).

**Table 3:** The description of significant SNPs and candidate genes for seven carcass traits

Trait^a^	SNP rs^b^	Location (bp)^c^	*p*-value^d^	–log_10_*p*^e^	MAF^f^	Allele	PEV (%)^g^	β(Se) ^h^	Gene ^i^	Distance (bp)^j^
DP	rs334674154	SSC10:9047896	5.80 × 10^− 7^	6.24	0.46	G/C	13.95	1.28(0.25)	***LYPLAL1***	Intron
	rs81321697	SSC10:42821846	2.19 × 10^− 6^	5.66	0.46	C/A	9.88	–1.31(0.27)	*EPC1*	D_13,439
	rs693030537	SSC10:42821848	2.63 × 10^− 6^	5.58	0.44	A/G	10.52	–1.28(0.27)	*EPC1*	D_13,437
	rs343827180	SSC10:42821905	8.77 × 10^− 7^	6.06	0.48	A/T	10.40	–1.35(0.27)	*EPC1*	D_13,380
	rs1109743164	SSC14:133704571	9.48 × 10^− 6^	5.02	0.08	T/G	7.78	–2.21(0.48)	NA	NA
		SSC14:133704611	7.90 × 10^− 6^	5.10	0.08	T/C	7.82	–2.23(0.48)	NA	NA
		AEMK020004 00.1:3892	8.68 × 10^− 6^	5.06	0.07	G/A	6.45	2.45(0.55)	NA	NA
RIB	rs708233859	SSC9:29825285	2.14 × 10^− 6^	5.67	0.25	A/G	3.01	0.33(0.07)	NA	NA
		SSC4:38814467	8.77 × 10^− 7^	6.06	0.05	A/G	10.79	–0.60(0.12)	*RPL30*	U_93,687
									***MATN2***	Intron
ST	rs330806505	SSC4:7108760	9.67 × 10^− 6^	5.01	0.32	G/A	7.98	0.05(0.01)	***ZFAT***	Intron
	rs320372958	SSC15:35899597	6.27 × 10^− 6^	5.20	0.41	C/T	8.98	–0.04(0.01)	*ENSSSCG00* *000044137*	D_94,956
	rs340678157	SSC15:35899625	2.71 × 10^− 6^	5.57	0.42	C/T	9.77	–0.04(0.01)	*ENSSSCG00* *000044137*	D_94,928
	rs328617466	SSC7:51823917	3.28 × 10^− 6^	5.48	0.09	C/T	12.40	0.09(0.02)	*HDGFL3*	U_1,539
									*TM6SF1*	Intron
									*BTBD1*	D_53,437
	rs318262569	SSC4:56089144	2.54 × 10^− 6^	5.60	0.29	A/T	**16.17**	0.06(0.01)	*ZBTB10*	D_46,889
									*ZNF704*	U_84,835
	rs325200532	SSC13:188457884	1.66 × 10^− 6^	5.78	0.07	C/T	7.40	0.11(0.02)	NA	NA
CSL	rs324898647	SSC8:7830994	7.80 × 10^− 6^	5.11	0.36	A/G	10.69	2.54(0.55)	NA	NA
	rs340085518	SSC8:7831068	8.59 × 10^− 6^	5.07	0.11	A/G	10.41	3.98(0.87)	NA	NA
	rs322537544	SSC8:8263513	9.87 × 10^− 6^	5.01	0.31	C/T	4.19	–2.77(0.62)	*ENSSSCG00* *000039778*	U_9,422
	rs330152743	SSC5:10772879	7.98 × 10^− 6^	5.10	0.47	T/C	9.93	2.39(0.52)	*TMPRSS6*	U_67,399
									*TEX33*	Intron
									*TST*	U_13,256
									*KCTD17*	U_53,920
									*CSF2RB*	D_43,492
									*MPST*	U_23,148
	rs690857471	SSC7:36193764	2.23 × 10^− 7^	**6.65** ^*^	0.10	T/A	6.20	5.01(0.94)	NA	NA
		SSC18:52920094	4.78 × 10^− 6^	5.32	0.07	T/G	9.39	–4.71(1.01)	***INHBA***	U_19,127
CDL		SSC10:18327923	2.75 × 10^− 6^	5.56	0.09	A/C	12.63	4.71(0.99)	***SMYD3***	Intron
		SSC18:52920094	4.45 × 10^− 7^	**6.35** ^*^	0.07	T/G	9.39	–5.38(1.04)	***INHBA***	U_19,127
	rs338797354	SSC4:122997439	8.21 × 10^− 6^	5.09	0.13	C/T	3.37	–3.83(0.84)	*ARHGAP29*	D_88,483
									*ABCD3*	U_44,182
LEW		SSC9:12068165	8.84 × 10^− 6^	5.05	0.08	G/A	12.83	–0.19(0.18)	***PAK1***	Intron
									*AQP11*	Intron
									*RSF1*	D_53,660
	rs708657805	SSC9:12068393	2.69 × 10^− 6^	5.57	0.08	A/G	13.41	–0.21(0.18)	***PAK1***	Intron
									*AQP11*	Intron
									*RSF1*	D_53,432
	rs324713326	SSC3:15128630	8.79 × 10^− 6^	5.06	0.09	G/A	6.68	–0.16(0.18)	NA	NA
	rs341980415	SSC3:27538828	8.34 × 10^− 6^	5.08	0.07	A/G	13.36	–0.02(0.20)	*XYLT1*	U_45,936
	rs337711004	SSC3:74664280	5.14 × 10^− 6^	5.29	0.32	A/G	9.79	0.26(0.10)	*ENSSSCG00* *000045421*	U_32,729
									*ETAA1*	D_56,789
LET	rs1113904511	SSC2:5773845	1.64 × 10^− 6^	5.79	0.27	G/A	7.51	–0.05(0.09)	*C11orf80*	U_76,046
									*RBM14*	Intron
									*RBM4B*	Intron
									***ACTN3***	D_85,000
									***SPTBN2***	D_34,428
									*CCS*	D_45,626
									*CTSF*	D_81,266
									*ENSSSCG00* *000040263*	D_42,630
									*CCDC87*	D_59,619
	rs1112016337	SSC4:27406931	1.45 × 10^− 6^	5.84	0.10	A/G	12.19	0.16(0.14)	*ENSSSCG00* *000050037*	Intron

^a^ DP, dressing percentage; RIB, number of ribs; ST, skin thickness; CSL, carcass straight length; CDL, carcass diagonal length; LEW, loin eye width; LET, loin eye thickness

^b^ SNP rs ID from Ensembl

^c^ Locations of the significant SNP according to the *Sus Scrofa* Build 11.1 assembly; *SSC*, *Sus Scrofa* chromosome

^d^ Minor Allele Frequency

^e^ Genome-wide significant associations are underlined

^e, f^ *and** represented the 10% and 1% genome-wide significance, respectively

^g^ Phenotypic Variation Explained

^h^ The beta value and standard error for the allele effect

^i^ The gene located with 100 kb upstream and downstream of the significant SNP, NA represented no genes with 100 kb upstream and downstream of the significant SNP

^j^ U/D represented the gene located upstream or downstream of the SNP (Intergenic region), within represented the SNP located with the gene

### DP, RIB, and ST

GWAS results and candidate genes for DP, RIB, and ST are showed in Table 3; Fig. 1.

On SSC10, four SNPs were significantly related to DP. Among, the rs334674154 was located in the lysophospholipase like 1 (*LYPLAL1*) gene. Another three adjacent SNPs, including rs81321697, rs693030537 and rs343827180, were located 13.4 kb downstream of the enhancer of polycomb homolog 1 (*EPC1*) gene. For RIB, the significant SNP (SSC4:38814467) was located with the matrilin-2 (*MATN2*) gene.

For ST, a total of six significant SNPs were detected on SSC4, SSC7, SSC13 and SSC15. Among, the rs330806505 was located within the zinc finger protein ZFAT (*ZFAT*) gene. The rs318262569 was located 46.9 kb downstream and 84.8 kb upstream of zinc finger and BTB domain containing 10 (*ZBTB10*) and zinc finger protein 704 (*ZNF704*), respectively.

### CSL, CDL, LEW and LET

GWAS results and candidate genes for CSL, CDL, LEW and LET are showed in Table 3; Fig. 2.

Interestingly, one significant SNP (SSC18:52920094) were found to be associated with CSL and CDL. The SNP was located 19.1 kb upstream of the inhibin beta A subunit (*INHBA*) gene. Additionally, the SNP SSC10:18327923 was significantly associated with CDL, which was located with the SET and MYND domain containing 3 (*SMYD3*) gene.

Furthermore, a total of five significant SNPs were detected to be related to LEW. Among, two adjacent genes (SSC9:12068165 and rs708657805) on SSC9 were located with p21 (RAC1) activated kinase 1 (*PAK1*) and aquaporin 11 (*AQP11*). For LET, one significant SNP (rs1113904511) on SSC2 was located 34.4 kb and 85.0 kb downstream of spectrin beta chain, non-erythrocytic 2 (*SPTBN2*) and alpha-actinin-3 (*ACTN3*), respectively.

### Comparison with previously mapped QTL in pigs

Based on SNP and QTL locations, the Pig QTL Database (Pig QTLdb, https://www.animalgenome.org/cgi-bin/QTLdb/SS/index, accessed on 25 April 2023) was searched to see if any of the QTLs connected to carcass traits in the study replicate any other QTLs that have been previously identified. In total, 30 SNPs significantly associated with carcass traits were identified, of which 9 SNPs were located in previously reported QTL regions in pigs. The remaining 21 SNPs had not been incorporated into any QTLs linked to carcass traits that had been previously reported. Interestingly, two QTLs on SSC10 (42.82–42.82 Mb, 59-bp) and SSC8 (78.31–82.64 Mb, 4.33-Mb) were found to be associated with DP and CSL, respectively. The results of QTLs comparison are shown in Table S3.

### Go annotation and functional enrichment analysis of candidate genes

The results of GO annotation showed that *INHBA* was involved in the negative regulation of cell proliferation. *SMYD3* participated in myotube cell development and negative regulation of protein kinase activity. *PAK1* participated in mitotic cell cycle. GO annotation results of other genes are shown in Table S4.

Furthermore, six GO terms including two BP terms (transsulfuration, and positive regulation of transcription from RNA polymerase II promoter), two MF terms (thiosulfate sulfurtransferase activity, and cullin family protein binding), one CC term (nucleoplasm) and two KEGG pathways (sulfur relay system, and sulfur metabolism) were significantly enriched (*p* < 0.05) (Table S5).

### Association analysis between SNP marker genotypes and carcass traits

The association analysis between candidate SNPs genotypes and carcass traits was performed. The result revealed that the genotype (G–A) of SNP rs330806505, presented significant associated with ST (*p* < 0.05). The allele G was favorable for thinner ST on rs330806505. Two homozygous genotypes of other SNPs presented extremely significant associated with corresponding traits (*p* < 0.01). Table 4 displays the genotype effects as well as additive and dominant effects.

## Discussion

### QTLs identified for carcass traits

A total of 30 SNPs were identified as significant for seven carcass traits investigated, of which 9 SNPs had located in previously reported QTLs for pig carcass traits. Three adjacent SNPs on SSC10 (59-bp internal) were associated with DP, which was located in a previously reported QTL region related to DP [20]. Additionally, three SNPs on SSC8 (78.31–82.64 Mb, 4.33-Mb) were found to be associated with CSL, which was located in a previously reported QTL region related to CSL [21].

### Candidate genes for DP, RIB, and ST

On SSC10, four SNPs were significantly related to DP. Among the SNPs, the rs334674154 was located in the *LYPLAL1* gene. Two homozygous genotypes of the SNP presented significant association with DP (*p* < 0.01). The DP of the CC genotype was 2.82% higher than that of GG (Table 4). The results of GO annotation revealed that *LYPLAL1* has hydrolase activity. Studies have found that common variants within or near the *LYPLAL1* gene are related to a variety of human metabolic characteristics, including obesity and fatty liver. Knockout of the *LYPLAL1* gene in mice would not lead to any significant phenotypic or metabolic physiological abnormalities in mice [22]. A recent study showed that the knockout of *LYPLAL1* gene could lead to the reduction of AKT2 phosphorylation and glucose uptake in human adipocytes induced by insulin [23]. Another three adjacent SNPs (rs81321697, rs693030537 and rs343827180), were located 13.4 kb downstream of the *EPC1* gene. The *EPC1* gene was found to be involved in skeletal muscle differentiation [24].

For RIB, the significant SNP (SSC4:38814467) was located with the *MATN2* gene. Two homozygous genotypes of the SNP presented significant association with RIB (*p* < 0.01). The RIB of the AA genotype was 1.1 more than that of GG genotype (Table 4). *MATN2* gene encodes matrix protein 2 (MATN2), an extracellular matrix (ECM) protein. ECM plays an important role in differentiation, maintenance and remodeling of tissues during development and regeneration. As a multiadhesion adaptor protein, MATN2 interacts with other ECM proteins and integrins to promote neurite outgrowth, Schwann cell migration, neuromuscular junction formation, skeletal muscle and liver regeneration and skin wound healing, and can also regulate multiple signal transduction pathways, including transforming growth factor beta, bone morphogenetic protein 7 and Smad signaling pathway, which are critical for muscle tissue regeneration [25]. Zhang et al. [26] found that *MATN2* gene was widely distributed in many connective tissues of mice, including tracheal cartilage, developing bone and adult bone. Compared with normal healthy tissues, the expression level of *MATN2* in developing articular cartilage tissue of osteoarthritis was significantly increased. This study suggested that *MATN2* might play an important role in the communication between matrix and matrix and between matrix and cells, and it could be used as a potential biomarker of early osteoarthritis of articular cartilage. In view of the extensive expression of *MATN2* in bone tissue and the role of its coding protein in tissue regeneration and osteoarthritis, we believe that *MATN2* could be a potential candidate gene for RIB.

GWAS results showed that two SNPs on SSC4 were significantly associated with ST, and these two loci were located near zinc finger protein gene, in which the SNP rs330806505 was located within the *ZFAT* gene. Genotype (G–A) of the SNP presented significant association (*p* < 0.05) with ST. The ST of genotype GG of SSC14:42805887 was 0.37 mm and 0.95 mm thinner than that of GA and AA, respectively. The rs318262569 was located 46.9 kb downstream and 84.8 kb upstream of *ZBTB10* and *ZNF704*, respectively. The results of GO annotation showed that *ZBTB10* and *ZNF704* had the function of metal ion binding. Zinc finger protein (ZFP) refers to a class of proteins that contain short, stable proteins that can self fold to form a “finger” structure by binding zinc^2+^(Zn^2+^). Zinc is a cofactor for more than 1,000 kinds of enzymatic reactions [27, 28], and it is necessary for more than 2,000 kinds of transcription factors [29]. Therefore, zinc is associated with a variety of organic activities, such as development, differentiation and cell growth. The functions of ZFP have DNA interaction, RNA packaging, transcriptional activation, apoptosis regulation, protein folding and assembly, and lipid binding [30–32]. About 10% of human proteins can bind with zinc [33]. Skin is the third highest zinc content tissue in the body [34]. Zinc plays a very important role in skin. Keratinocyte (KC) in the skin accounts for about 97% of the skin epidermis. It can be divided into four layers according to the degree of differentiation and keratinization, including the basal layer, acanthous layer, granular layer and cuticle. Zinc is most abundant in the acanthous layer [35]. In vitro experiments showed that exposure of HaCaT KCs (HaCaT keratinocytes) to non-toxic concentrations of zinc could promote their survival and proliferation. A large number of experiments proved that zinc is necessary for keratinocytes to proliferate [36–38]. In addition, mutations or disorders of zinc transporters and zinc deficiency are closely related to human related skin diseases (including acquired acrodermatitis, necrotic transitional erythema, pellagra, etc.) [39]. To sum up, ZFP plays an important role in the proliferation and differentiation of skin by combining with zinc. Therefore, zinc finger protein genes *ZFAT*, *ZBTB10* and *ZNF704* could be considered as potential candidate genes for pig ST.

### Candidate genes for CDL and CSL

Interestingly, one significant SNP (SSC18:52920094) was found to be associated with CSL and CDL. The SNP was located 19.1 kb upstream of the *INHBA* gene. Two homozygous genotypes of the SNP presented significant association with CSL and CDL (*p* < 0.01). Genotype TT was 9.97 cm and 12.16 cm longer than genotype GG in CSL and CDL, respectively (Table 4). GO annotation results revealed that the gene had the function of growth factor activity and transforming growth factor beta receptor binding, and was involved in the negative regulation of cell proliferation. *INHBA* gene encodes INHBA, which is a member of TGF-β superfamily. The known TGF-β signal pathway is involved in cell proliferation and apoptosis [40], which is very important for female reproductive process [41]. A large number of studies have proved that *INHBA* gene had a significant impact on the fecundity of livestock [42–44]. At present, there is no report indicating that *INHBA* gene is associated with carcass length. Additionally, the SNP SSC10:18327923 was significantly associated with CDL, which was located with the *SMYD3* gene. Two homozygous genotypes of the SNP presented significant association with CDL (*p* < 0.01). The CDL of genotype CC was 8.3 cm longer than that of genotype AA (Table 4). GO annotation results showed that *SMYD3* gene was involved in myotube cell development and negative regulation of protein kinase activity. *SMYD3* encodes SMYD3, a histone methyltransferase, which has been found to be related to muscle volume and skeletal muscle atrophy [45]. The study found that *SMYD3* gene might be a potential candidate gene for hind leg muscle atrophy of canine hip dysplasia [46]. Therefore, *SMYD3* gene could be considered as a potential candidate gene for CDL.

### Candidate genes for LEW and LET

Two adjacent SNPs (SSC9:12068165 and rs708657805) on SSC9 were detected to be related to LEW. Two homozygous genotypes of these two SNPs presented significant association with LEW (*p* < 0.01). The LEW of genotype AA for two SNPs was 0.79 cm and 0.81 cm wider than that of GG genotype, respectively (Table 4). These two loci were located in two overlapping genes, including *PAK1* and *AQP11*. GO annotation results showed that *PAK1* gene had the function of ATP binding and participated in mitotic cell cycle. PAK1 is a member of PAK family, which includes 6 members and is divided into 2 groups. The first group includes PAK1, PAK2 and PAK3, and the second group includes PAK4, PAK5 and PAK6. Among them, PAK1 is expressed in myoblasts and specifically activated during mammalian myoblastic differentiation. PAK1 is a regulator of myoblast differentiation in vitro and in vivo, and participates in the promyogenic lNcad/Cdo/Cdc42 signal pathway [47]. Studies have found that *PAK1* and *AQP1* were candidate genes for cooking loss [48]. In conclusion, *PAK1* gene was more likely to be a candidate gene for the loci.

For LET, one significant SNP (rs1113904511) on SSC2 was located 34.4 kb and 85.0 kb downstream of *SPTBN2* and *ACTN3*, respectively. GO analysis found that *SPTBN2* gene had the function of actin binding and structural component of cytoskeleton (Table 4). De León et al. [49] believed that *SPTBN2* gene might have a negative impact on the muscle development of cattle. Furthermore, GO analysis results showed that *ACTN3* gene had the function of calcium ion binding, and participated in the regulation of the force of skeletal muscle contraction, positive regulation of skeletal muscle tissue growth, and positive regulation of skeletal muscle fiber development. *ACTN3* encodes α-actinin-3 (ACTN3), which is only expressed in type II muscle fibers (fast, glycolytic), while α-actinin-2 presents in all skeletal muscle fibers [50, 51]. α-actinin performs static functions in skeletal muscle, maintains myofibril network and coordinates muscle contraction [51, 52]. Studies have found that the loss of *ACTN3* gene function could change muscle metabolism in mice [53]. Since *ACTN3* is only distributed in type II muscle fibers and its function in muscle, *ACTN3* gene should be considered as a strong candidate gene for loin eye traits, including LET trait.

**Table 4:** Effect of the genotypes on several carcass traits

Trait^a^	SNP rs^b^	Location (bp)^c^	Genotype^d^	*N* ^e^	Phenotype value^f^	Additive effect	Dominance effect	Gene
DP (%)	rs334674154	SSC10: 9,047,896	GG	69	73.77 ± 0.34 ^B^	1.41	-0.68	*LYPLAL1*
			GC	59	74.50 ± 0.37 ^B^			
			CC	55	76.59 ± 0.38 ^A^			
			NN	40				
RIB		SSC4: 38,814,467	AA	166	14.10 ± 0.04 ^A^	0.55	0.01	*MATN2*
			AG	16	13.56 ± 0.13 ^B^			
			GG	2	13.00 ± 0.38 ^B^			
			NN	27				
ST (mm)	rs330806505	SSC4: 7,108,760	GG	94	3.25 ± 0.11 ^cB^	0.48	-0.11	*ZFAT*
			GA	65	3.62 ± 0.14 ^bAB^			
			AA	26	4.20 ± 0.22 ^aA^			
			NN	38				
CSL (cm)		SSC18: 52,920,094	TT	174	97.47 ± 0.43 ^A^	4.99	0.30	*INHBA*
			TG	25	92.78 ± 1.13 ^B^			
			GG	2	87.50 ± 3.99 ^B^			
			NN	22				
CDL (cm)		SSC18: 52,920,094	TT	174	84.16 ± 0.46 ^A^	6.08	1.22	*INHBA*
			TG	25	79.30 ± 1.23 ^B^			
			GG	2	72.00 ± 4.33 ^B^			
			NN	22				
		SSC10: 18,327,923	AA	158	82.70 ± 0.45 ^B^	4.15	1.48	*SMYD3*
			AC	26	88.33 ± 1.11 ^A^			
			CC	3	91.00 ± 3.27 ^A^			
			NN	36				
LEW (cm)		SSC9: 12,068,165	GG	162	8.13 ± 0.07 ^B^	0.90	-0.48	*PAK1*
			GA	13	8.55 ± 0.25 ^B^			
			AA	7	9.92 ± 0.34 ^A^			
			NN	39				
	rs708657805	SSC9: 12,068,393	AA	164	8.11 ± 0.07 ^B^	0.91	-0.41	*PAK1*
			AG	12	8.61 ± 0.26 ^B^			
			GG	7	9.92 ± 0.34 ^A^			
			NN	38				

^a^ DP, dressing percentage; RIB, number of ribs; ST, skin thickness; CSL, carcass straight length; CDL, carcass diagonal length; LEW, loin eye width

^b^ SNP: rs ID from Ensembl

^c^ SSC: *Sus scrofa* chromosome; Locations of the significant SNP according to the *Sus scrofa* Build 11.1 assembly

^d^ NN represents no genotype

^e^ N: Number of pig accessions

^f^ Different capital letters indicate an extremely significant difference (*p* < 0.01), and different lowercase letters indicate a significant difference (*p* < 0.05)

However, more pig populations must be used to verify these SNPs and candidate genes in future studies, and more pig biological tests must be carried out to confirm the roles and functions of these loci and genes.

## Conclusions

In conclusion, we conducted a GWAS based on SLAF-seq for seven carcass traits in 223 four-way intercross pigs using MLM. A total of 30 significant SNPs, two QTLs on SSC8 and SSC10, and 11 candidate genes (*LYPLAL1*, *EPC1*, *MATN2*, *ZFAT*, *ZBTB10*, *ZNF704*, *INHBA*, *SMYD3*, *PAK1*, *SPTBN2*, and *ACTN3*) were identified as being associated with carcass traits of pigs. Overall, our study provided new evidences that multiple genes were involved in regulating carcass traits in pigs. These SNPs and corresponding candidate genes served as a biological foundation for improving carcass quality in swine breeding.

## Materials and methods

### Animals, phenotypic collection and statistical analysis

As described previously [17–19], a population of four-way crossbred pigs was set up. To put it briefly, a total of 223 four-way crossbred animals (108 males and 115 females, DSYLS) from 59 litters were created by mating 7 hybrid boars (Duroc × Saba, DS) and 37 hybrid sows (Yorkshire × (Landrace × Saba), YLS). Information statistics of for each pig accession and each litter showed in Table S6 and Table S7. The diets and environmental conditions for all test pigs were kept the same, and they all had unlimited access to food and water. 223 pigs were injected 10% potassium chloride injection rapidly intravenously at a rate of 0.3 ~ 0.5 milliliters per kilogram of body weight when they weighed 105.25 ± 15.75 kg on average. All animals were slaughtered after euthanasia. During this time, ear tissue samples were gathered.

Seven carcass traits, including DP, RIB, ST, CSL, CDL, LEW and LET were measured according to the method of “Technical regulation for testing of carcass traits in lean-type pig” (NYT825-2004).

The SAS 9.4 (SAS Institute, Inc., Cary, NC) MEANS procedure was used to generate descriptive statistics for seven carcass traits under study, including the total number, minimum, maximum, mean, standard deviation, and coefficient of variation. Using the R package “ggpubr”, the sample distribution was visualized as a frequency distribution histogram. The phenotypic correlation study was conducted using the R function “PerformanceAnalytics”. Using GCTA software, genetic correlations for seven carcass traits were calculated [54].

### SLAF library construction and high-throughput sequencing

As described previously, molecular markers throughout the entire pig genome were generated and SLAF library were constructed [17–19]. Meanwhile, the control genome (Oryza sativa spp. japonica; 374.30 Mb; http://rapdb.dna.affrc.go.jp/) was used to verify the reliability of the experimental process. Ultimately, SLAF-seq for each individual was conducted on an Illumina HiSeq 2500 platform (Illumina, Inc., San Diego, CA, USA) at Beijing Biomarker Technologies Corporation in Beijing, China.

### Data processing and SNP calling

As described previously, data processing and SNP calling were further constructed [17–19]. Briefly stated, Dual-Index software [55] was used to further analyze the raw SLAF-seq data in order to produce the raw paired-end sequencing reads for each accession. Then, using BWA software [56], raw paired-end reads were aligned to the *Sus scrofa* reference genome (Sscrofa 11.1_102). Afterward, polymorphic SLAF tags were obtained. SNP loci were discovered based on data from polymorphic SLAF tags, primarily using GATK [57]. Clean reads that matched to the reference genome were used as the foundation for local realignments, base recalibration, and SNP detection with GATK [57]. The accuracy of the SNPs found using GATK was ensured by utilizing SAMtools [58]. The overlap of SNPs discovered by both GATK and SAMtools were selected as the reliable collection of SNPs to be submitted to the following analysis. Using PLINK 2 [59], a total of 227, 921 SNPs were acquired for further research by filtering for minor allele frequency (MAF: 0.05) and integrity (int: 0.8).

### Population structure analysis and genome-wide association study (GWAS)

Population structure analysis was performed using ADMIXTURE software [60]. 227,921 total filtered SNPs detected from 223 accessions were used for GWAS. The mixed linear model (MLM) of GEMMA software [61] was employed for association analysis between carcass traits and reliable SNP markers. The MLM formula of GEMMA software was as follows:

1 $$y = {\text{W}}\alpha + {\text{X}}\beta + {\text{Z}}\mu + \varepsilon$$

Where *y* was a *n*×1 vector of phenotype in crossbred pigs, X was a *n*×1 vector of marker genotypes, W was the matrix of population structure calculated by the ADMIXTURE software [60], and Z was the matrix of the kinship relationship calculated using GCTA software [62]. α was the vector of fixed effects; β was the marker effects; µ was random effects and ε was the vector of residuals. The association result could then be determined for each variation site. Bonferroni correction (BC) approach [61] was used for multiple tests in the study. Markers with adjusted − log10 (*p*) > 5 (control threshold) were deemed to be significant SNPs for carcass traits of interest [17–19]. Based on the number of filtered SNPs (*n* = 227,921), the threshold *p*-value for genome-wide 1% and 10% significance were 4.39 × 10^− 8^ (0.01/227,921) and 4.39 × 10^− 7^ (0.1/227,921), respectively. Using the R package “qqman”, the manhattan and quantile-quantile (Q-Q) plots of GWAS were drawn.

### Identification, annotation and functional enrichment analysis of candidate genes

Based on the previous references [63, 64], the genes within 100 kb upstream or downstream of significant associated SNPs were deemed as potential candidate genes for carcass traits. The relevant information on these potential genes was downloaded from the Ensembl Sscrofa11.1 database (www.ensembl.org). Afterward, GO annotation of candidate genes was conducted using Gene Ontology Consortium (http://geneontology.org). The database for annotation, visualization, and integrated discovery (DAVID v6.8, https://david.ncifcrf.gov/) was used to perform GO and KEGG enrichment analyses based on genes that were 100 kb upstream and downstream of significant SNPs. A threshold p-value of 0.05 was used to determine whether GO terms and KEGG pathways were significantly enriched.

#### Association analysis between SNP marker genotypes and growth traits

Proc GLM in SAS 9.4 (SAS Institute, Inc., Cary, NC) was used to estimate the associations between SNP marker genotypes and carcass traits. Additive genetic effects were calculated by comparing the two homozygous genotypes in pairs, and the dominance effects were computed as the deviation of the heterozygote effect from the mean of the two homozygous genotypes.

### Electronic supplementary material

Below is the link to the electronic supplementary material.

Supplementary Material 1

Supplementary Material 2

Supplementary Material 3

Supplementary Material 4

Supplementary Material 5

Supplementary Material 6

Supplementary Material 7

Supplementary Material 8

Supplementary Material 9

Supplementary Material 10

Supplementary Material 11

## Data Availability

The genome sequencing raw data was deposited in NCBI’s SRA database https://www.ncbi.nlm.nih.gov/bioproject/?, Accession: PRJNA842083).

## Abbreviations

GWAS: Genome-wide association study
SLAF-seq: Specific-locus amplified fragment sequencing
SNP: Single nucleotide polymorphism
QTL: Quantitative trait loci
DP: Dressing percentage
RIB: Number of ribs
ST: Skin thickness
CSL: Carcass straight length
CDL: Carcass diagonal length
LEW: Loin eye width
LET: Loin eye thickness
SSC: Sus scrofa chromosome
MAF: Minor allele frequency
PVE: Phenotypic variation explained
Q-Q plot: Quantile-quantile plot
MLM: Mixed linear model
GO: Gene ontology
WGS: Whole-genome sequencing
GC: Guanine-cytosine
