# Supplementary figures and images for: Genome-wide identification of quantitative trait loci and candidate genes for seven carcass traits in a four-way intercross porcine population
Source: BMC Genomics. 2024 Jun 10;25:582. doi: 10.1186/s12864-024-10484-y (PMC11165779; doi:10.1186/s12864-024-10484-y)

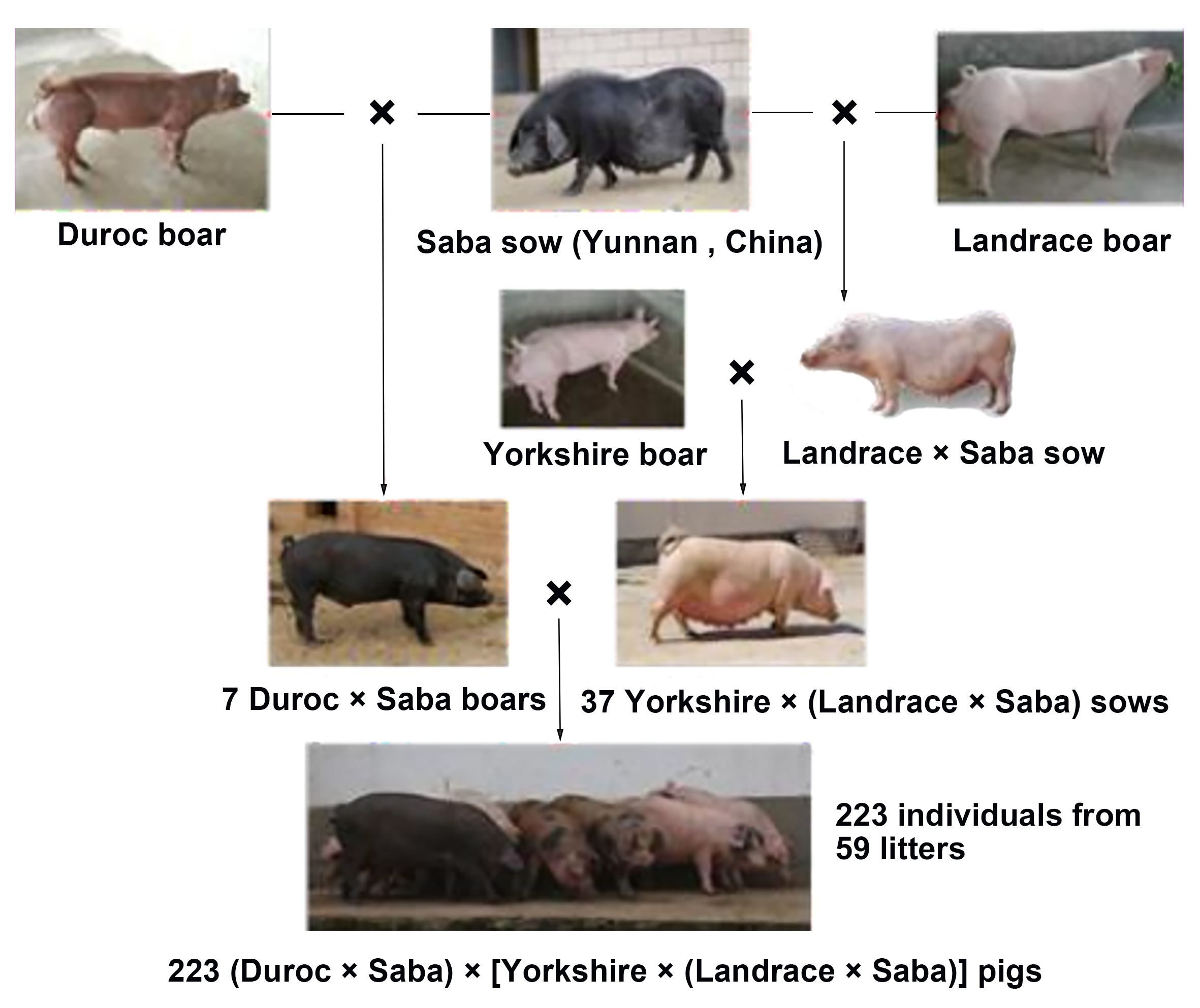

Supplement: Supplementary file 1 — Supplementary Material 1 [file 12864_2024_10484_MOESM1_ESM.tif]

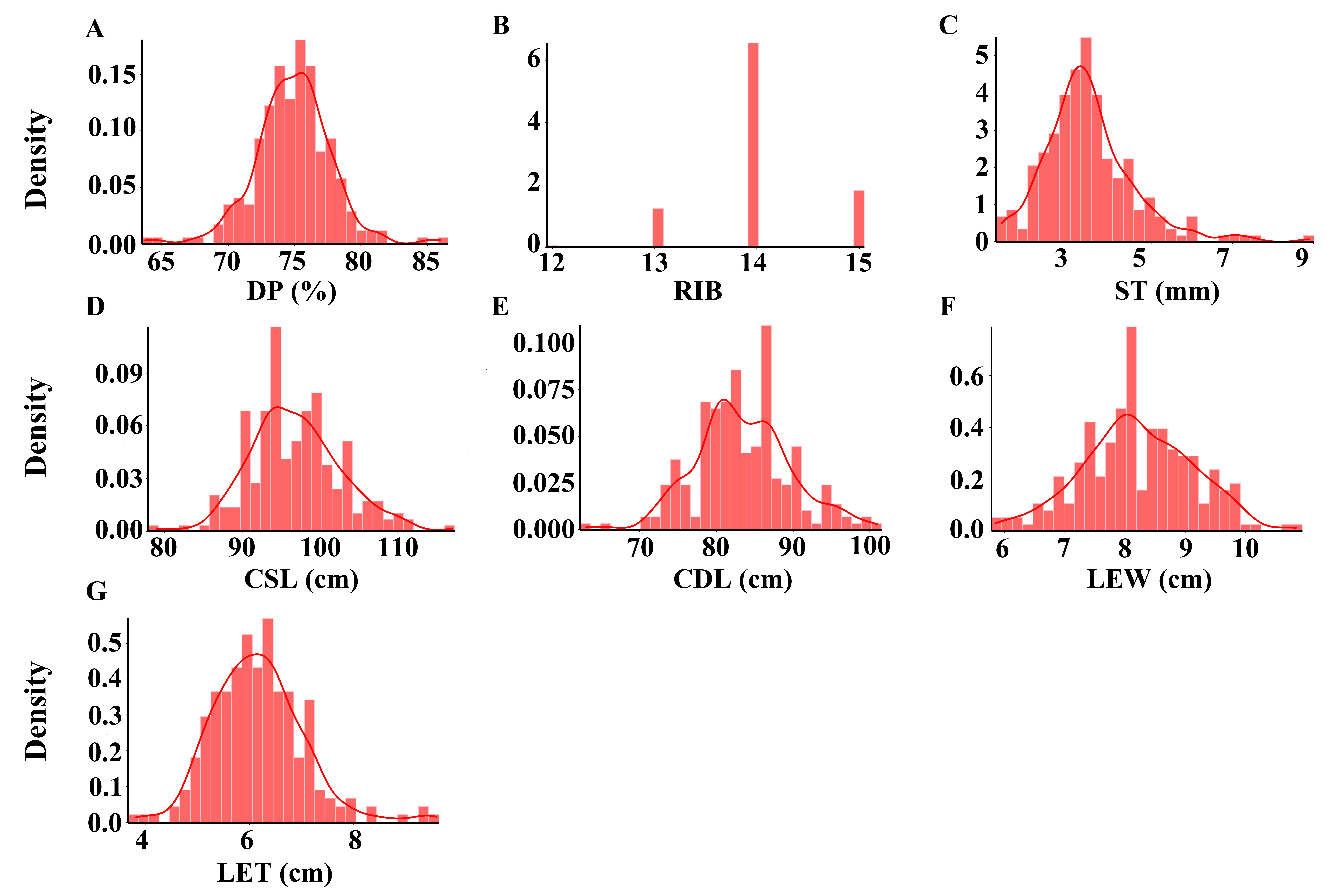

Supplement: Supplementary file 2 — Supplementary Material 2 [file 12864_2024_10484_MOESM2_ESM.tif]

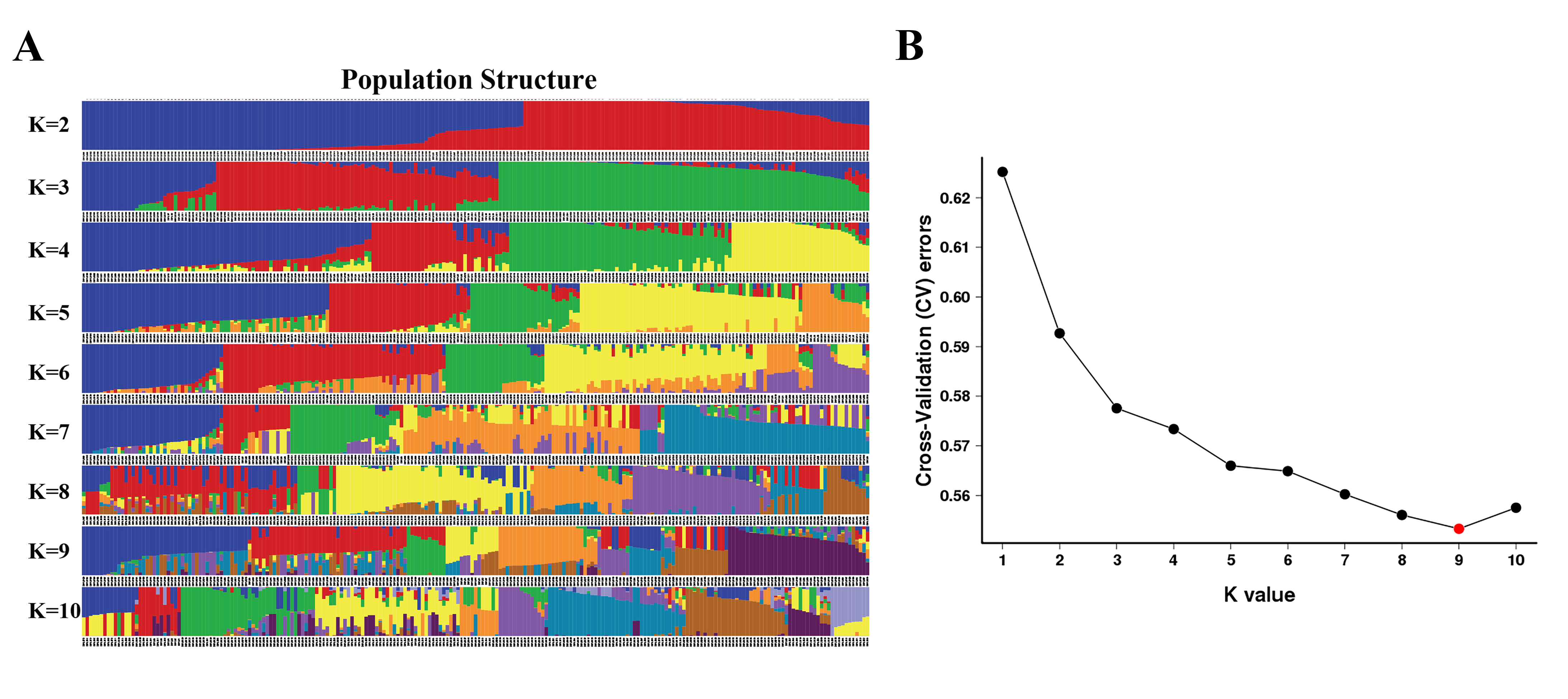

Supplement: Supplementary file 4 — Supplementary Material 4 [file 12864_2024_10484_MOESM4_ESM.tif]
